# Supplementary figures and images for: The C. elegans DSB-2 Protein Reveals a Regulatory Network that Controls Competence for Meiotic DSB Formation and Promotes Crossover Assurance
Source: PLoS Genet. 2013 Aug 8;9(8):e1003674. doi: 10.1371/journal.pgen.1003674 (PMC3738457; doi:10.1371/journal.pgen.1003674)

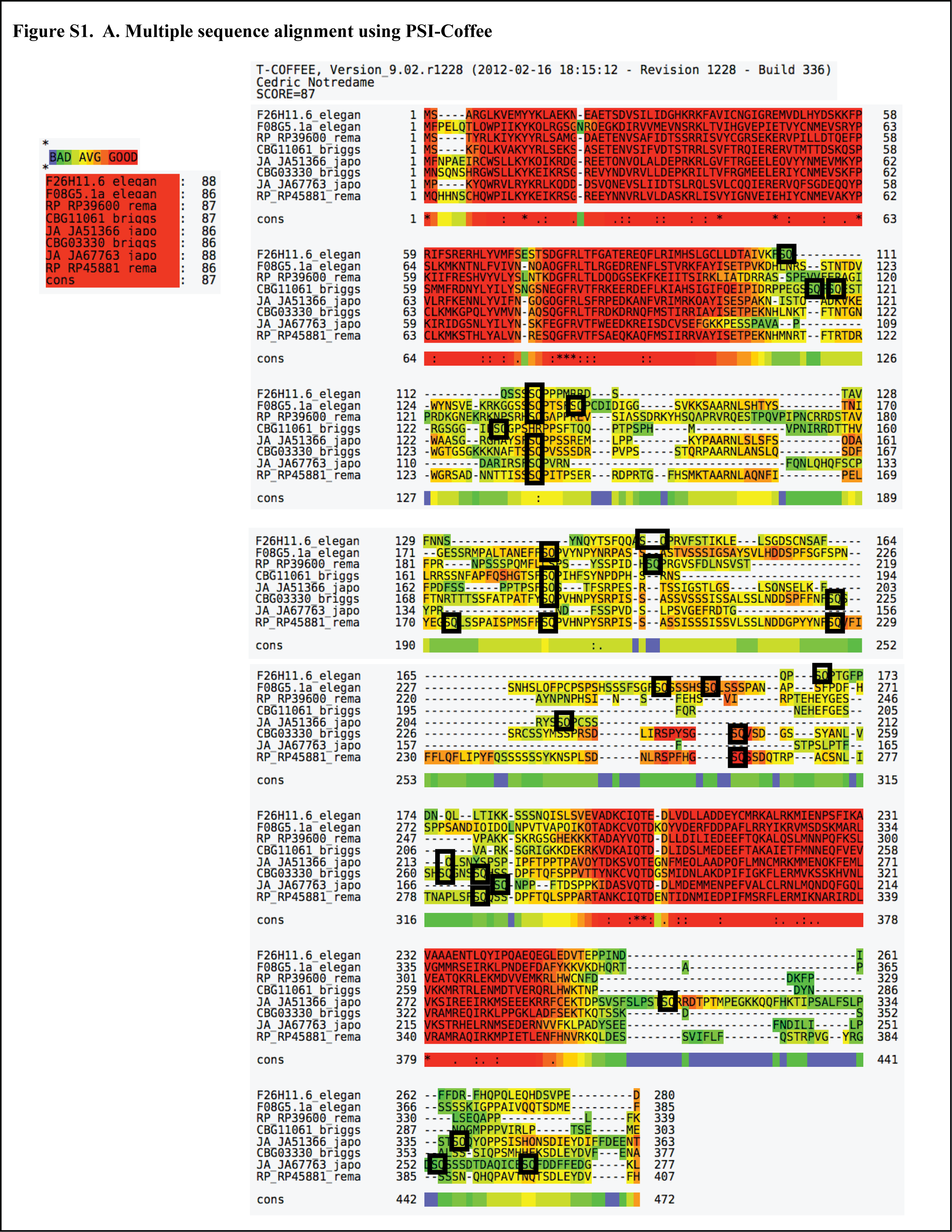

Supplement: Figure S1 — Multiple sequence alignment of DSB-2 family proteins. Multiple sequence alignment of DSB-2 family proteins produced by T-Coffee, a consistency-based aligner (a class known for increased accuracy, at the expense of slower speed) [58]. The alignment was produced in PSI-Coffee mode, recommended for alignment of remote homologues. The scores and associated color scheme represent consistency values, with the overall sequence score (top left) showing the relative fit of each sequence within the alignment. The red-colored residues represent reliably-aligned portions, while blue and green-colored stretches represent unreliable portions of the alignment. An asterisk (*) indicates positions that have a single, fully conserved residue. A colon (:) indicates conservation between amino acid groups of strongly similar properties (scoring >0.5 in the Gonnet PAM 250 matrix). A period (.) indicates conservation between amino acid groups of weakly similar properties (scoring ≤0.5 in the Gonnet PAM 250 matrix). The protein family shows two reliably aligned domains, corresponding to F26H11.6 (DSB-2) residues 1–103 and 195–251. These domains show some conserved stretches, most prominently a (D/E/Q) GFR (V/L) (T/S/L) motif, and a (I/V) QT (D/E) motif. These two domains are connected by a stretch containing several SQ residues, which are potential targets for phosphorylation, and are highlighted by black boxes. (TIF) [file pgen.1003674.s001.tif]

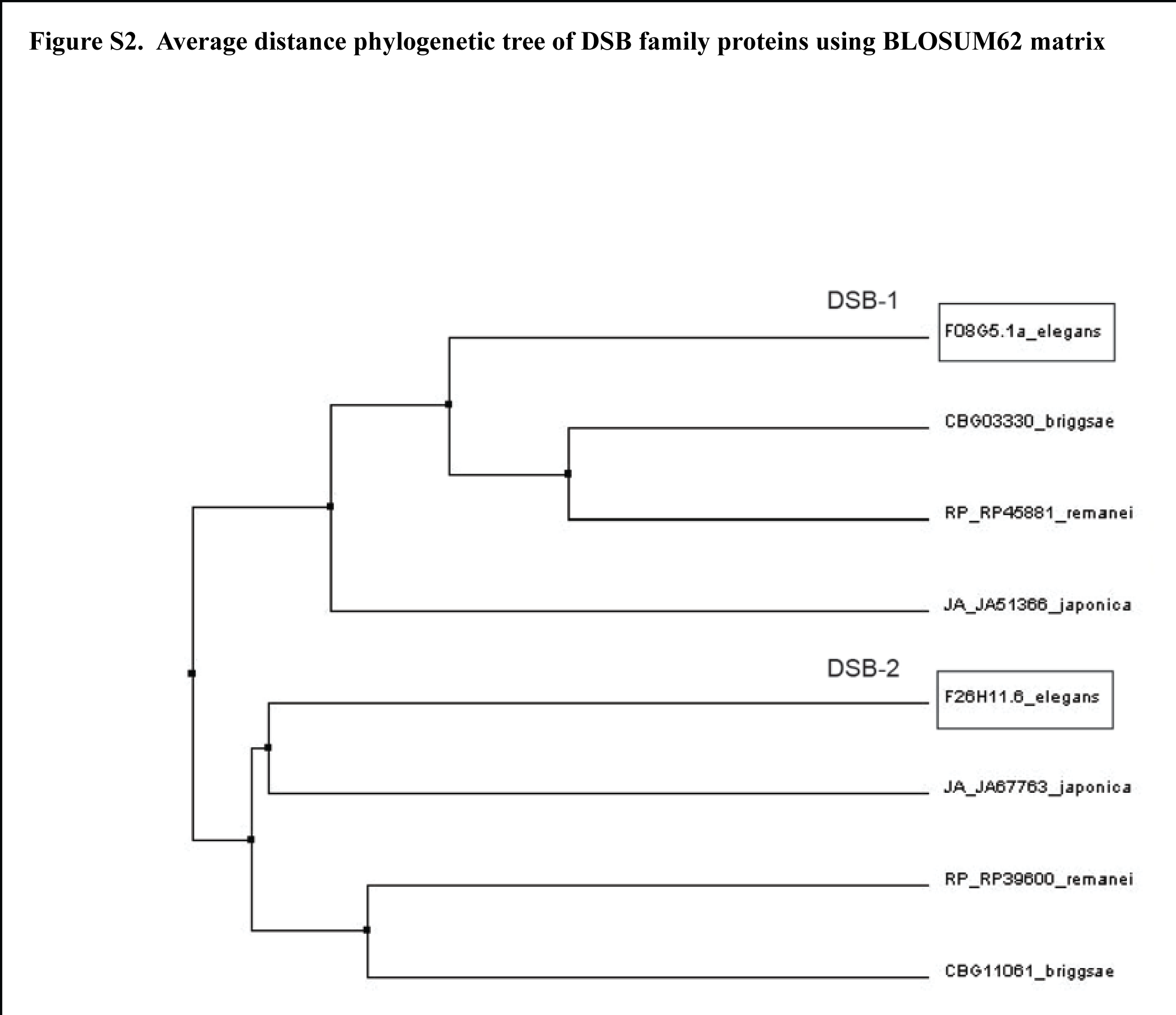

Supplement: Figure S2 — Phylogenetic tree of DSB-2 family proteins. Average distance phylogenetic tree of DSB-2 family proteins generated using BLOSUM62 matrix. This tree is based on the T-Coffee alignment. This protein family is highly divergent, with the two closest members showing only 51% identity (CBG03330_briggsae and RP45881_remanei). The F26H11.6 (DSB-2) protein shows <30% identity with any member of the family. Multiple sequence alignment and associated phylogenetic trees suggest that an early duplication event occurred before the species separated, and that the F26H11.6 branch is diverging more rapidly. The slightly more conserved F08G5.1 (DSB-1) branch reflects the evolutionary relationship of the species [59]. (TIF) [file pgen.1003674.s002.tif]
